# A Technique for Rapid Bacterial-Density Enumeration through Membrane Filtration and Differential Pressure Measurements

**DOI:** 10.3390/mi13081198

**Published:** 2022-07-28

**Authors:** Xinhui Shen, Ting Wei Teo, Tian Fook Kong, Marcos

**Affiliations:** School of Mechanical and Aerospace Engineering, Nanyang Technological University, Singapore 639798, Singapore; shenxh@ntu.edu.sg (X.S.); teotw@ntu.edu.sg (T.W.T.); tianfook@ntu.edu.sg (T.F.K.)

**Keywords:** bacterial enumeration, membrane filtration, pressure differential measurement, hydraulic resistance

## Abstract

In this article, we present a microfluidic technique for the rapid enumeration of bacterial density with a syringe filter to trap bacteria and the quantification of the bacterial density through pressure difference measurement across the membrane. First, we established the baseline differential pressure and hydraulic resistance for a filtration membrane by fully wetting the filter with DI water. Subsequently, when bacteria were infused and trapped at the pores of the membrane, the differential pressure and hydraulic resistance also increased. We characterized the infusion time required for the bacterial sample to achieve a normalized hydraulic resistance of 1.5. An equivalent electric-circuit model and calibration data sets from parametric studies were used to determine the general form of a calibration curve for the prediction of the bacterial density of a bacterial sample. As a proof of concept, we demonstrated through blind tests with *Escherichia coli* that the device is capable of determining the bacterial density of a sample ranging from 7.3 × 10^6^ to 2.2 × 10^8^ CFU/mL with mean and median accuracies of 87.21% and 91.33%, respectively. The sample-to-result time is 19 min for a sample with lower detection threshold, while for higher-bacterial-density samples the measurement time is further shortened to merely 8 min.

## 1. Introduction

The ability to determine the bacterial density in aqueous solution is crucial and desired in many bacteriological studies and industrial applications [1,2,3,4]. Among the methods devoted for enumerating sample bacterial density, the agar plate count is considered as the “gold standard” [5,6,7,8], in which a diluted bacterial solution is spread on an agar plate and the visible single-bacterial colonies formed after 24–48 h of incubation are counted [9,10,11]. However, the long incubation time and the fact that some bacteria are viable but not culturable [12,13] hinder the use of agar plate count to many experiments that require the knowledge of the density of the bacterial sample to proceed with subsequent experiments [14,15,16]. Furthermore, another major hurdle for agar plate count is in determining the dilution factor [17]. The number of colonies formed on the plate should ideally be between 20 and 200 colonies (or ~10^3^ CFU/mL) to avoid instances where the agar plate has too few or too many colonies to count. In order to achieve a more accurate and consistent estimation, one has to take the average colony-forming unit (CFU) for 5–10 agar plates for count averaging to mitigate sampling error or inconsistency. Therefore, for a bacteria sample of unknown concentration, one has to prepare far more agar plates with multiple dilution factors.

In a bid to reduce the sample-to-result time, many culture-independent techniques are developed to mitigate the need for cell culture or incubation [5,18,19]. To date, McFarland turbidity standards [20,21,22] and spectrophotometer measurements [23,24,25] are the two commonly adopted low-cost practices, which give rapid and reasonable estimations of high-density microbes (in the order of 10^8^ CFU/mL in less than 15 min, including spectrophotometer warm-up time) [26,27]. To detect low-density microbes, filtration-based methods through membrane and lab-on-chip devices offer great advantages in concentrating large sample volumes [28,29,30,31]. This, combined with fluorescence microscopy [32,33] or adenosine triphosphate (ATP) bioluminescence [34,35], pushes the detection limit to as low as 10^3^ CFU/mL in a few hours’ time. Nevertheless, similar to other advanced techniques such as nucleic acid-based [36] and immunological-based [18] methods, flow cytometry [37,38,39] and many biosensors [40,41,42,43,44,45], high accuracy and/or low detection limit are always associated with much more expensive cost for equipment, consumables and/or sophisticated labors [46,47]. In other words, a microbial enumeration technique that can rapidly enumerate bacterial density in a cost-effective manner is still lacking and remains to be desired.

In this work, we present a low-cost and rapid microfluidic technique based on membrane filtration and differential pressure measurements to determine bacterial density of samples ranging from 10^6^ to 10^8^ CFU/mL within 19 min. In order to determine the bacterial density of a given sample, we first have to characterize and establish the relationship between the bacterial density and change in the differential pressure across the filtration membrane. Bacteria are infused into the syringe filter after saturating the filter with DI water. As more and more bacteria are infused and trapped by the pores on the filtration membrane, the differential pressure across the membrane increases with time. We defined a parameter denoted as the termination time, *t*_0_, at an instance where the normalized pressure difference across the filtration membrane is increased to 1.5 times of that due to DI water. An equivalent electric-circuit model was developed to formulate the general form of a calibration curve for the prediction of the bacterial density of a bacterial sample.

As a proof of concept for our proposed method, we performed a parametric study with ten separate sets of experiments, each with *Escherichia coli* (*E. coli*) bacteria prepared at six different dilution factors. Based on results from these 60 runs, we obtained a calibration curve for the relative pressure difference in relation to the sample bacterial concentration with a remarkable goodness of fit R^2^ of 0.955 for *E. coli* cell concentration ranging from 10^6^ to 10^8^ CFU/mL. Note that this calibration is only required to be performed once per genus and species of bacteria type. While we demonstrated the use case scenario for *E. coli*, the calibration can be repeated for other bacteria or cells of interest such as *Salmonella*, *Staphylococcus* and *Bacillus subtilis*. Once the calibration curve is obtained, the bacterial density of a sample with unknown density can be determined. We performed a blind test consisting of 54 *E. coli* samples and demonstrated that the calibration curve is capable of determining the bacterial density in less than 19 min with mean and median accuracies of 87.21% and 91.33%, respectively. Samples of higher bacterial density (in the order of 10^8^ CFU/mL) reduce the detection time to as low as 8 min.

## 2. Materials and Methods

### 2.1. Device Schematic and Assembly

**Figure 1:**
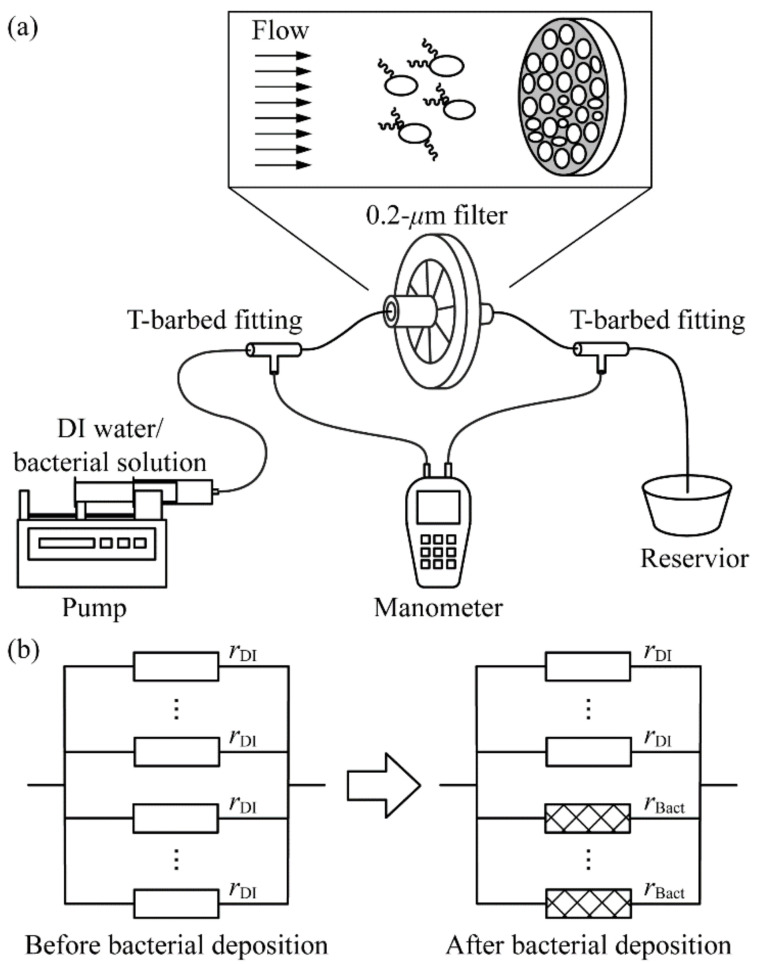
(**a**) Schematic diagram of the experiment setup. One end of a 0.2 µm filtration membrane is connected to a 50 mL syringe, the other end to a reservoir. The pressure difference across the filtration membrane is measured by a manometer. Inset illustrates that bacteria carried by background flow are trapped by the pores on the filtration membrane. (**b**) Equivalent parallel electric circuits of the filtration membrane (left panel) before and (right panel) after bacterial deposition. The averaged hydraulic resistances of a membrane pore before and after bacterial deposition are denoted by *r*_DI_ and *r*_Bact_, respectively.

The schematic diagram of our device is shown in Figure 1a. The setup comprises of a filtration membrane of 15 mm in diameter and 0.2 µm in pore size (431215, Corning Inc., Corning, NY, USA) connected to a 50 mL syringe (309653, BD, Franklin Lakes, NJ, USA) at the inlet and a reservoir at the outlet via Tygon tubing (Tygon E-3603, Saint-Gobain Performance Plastics, Akron, OH, USA). The syringe is driven by a syringe pump (Legato 210P, KD Scientific, Holliston, MA, USA) at a constant flow rate *Q*. The reservoir is open to the atmosphere. The pressure difference between up- and downstream of the filtration membrane, denoted by Δ*p*, is measured by a digital differential manometer (HD750, Extech Instruments, Nashua, NH, USA), which is connected to the tubing via two T-barbed fittings (Masterflex 50624-30, Avantor, Radnor Township, PA, USA).

### 2.2. Bacteria Sample Culture and Preparation

The bacterium *E. coli* (29947, ATCC, Manassas, VA, USA) was used to demonstrate the capabilities of our device. We cultured *E. coli* samples used for bacterial density enumeration by transferring a single colony from a streaked nutrient agar (Difco 213000, BD, Franklin Lakes, NJ, USA) to a culture tube (187261, Greiner, Frickenhausen, Germany) filled with 6 mL Nutrient Broth (Difco 234000, BD, Franklin Lakes, NJ, USA). The culture tube was then incubated at 37 °C and 250 rpm for 24 h. We filtered out large debris, if any, in the culture by passing the stock solution through three 5 µm filtration membranes (SLSV025LS, Merck Millipore, MA, USA) connected in series. After that, the density of filtered *E. coli* was determined via agar plate count by averaging the CFU counts from 10 agar plates. Results show that the *E. coli* density varies among different stocks but is in the order of 10^9^ CFU/mL. To eliminate the growth of bacterial cells over the course of the experiment, the bacteria in the filtered stock solution were thermally inactivated immediately after plating. This was carried out by putting the culture tube in 98 °C water bath for 25 min. Microscopic observation shows that the *E. coli* shape remained unchanged after thermal treatment.

### 2.3. Experiment Procedures for Obtaining the Calibration Curve for Bacteria Enumeration

Prior to bacterial-density enumeration, we used a four-step approach to establish and calibrate the relationship between the density of infused bacterial sample with the increase in the differential pressure. First, we filled the syringe with DI water and infused 16.5 mL into the filter at the infusing rate *Q* = 3 mL/min for three times. This was to calibrate the inherent pressure difference across the filter membrane without bacterial deposition. Second, we prepared six tubes of lower-density bacterial solution by diluting the filtered and inactivated bacterial stock solution with autoclaved DI water by 10, 20, 40, 100, 133 and 200 times. Third, we filled a new syringe with diluted bacterial solution and infused diluted *E. coli* samples into the filter at the same *Q*. We estimated that each experiment would take about 30 min. The experiment was repeated 10 times, so we obtained 60 sets of pressure readings in total. Last, an empirical formula was developed to find the bacterial density as a function of differential pressure. 

## 3. Results and Discussions

### 3.1. Establishing the Baseline Differential Pressure and Hydraulic Resistance of a Syringe Filter with DI Water

We note that the pore geometries and distributions of the filtration membranes may vary among one another, and thus, the pressure drop across different filtration membranes, Δ*p*, may vary. To quantify this variation, we adopted the hydraulic resistance [48], *R*, which measures Δ*p* at the flow rate *Q* and is defined as

(1) $$R=\frac{\Delta p}{Q}.$$

**Figure 2:**
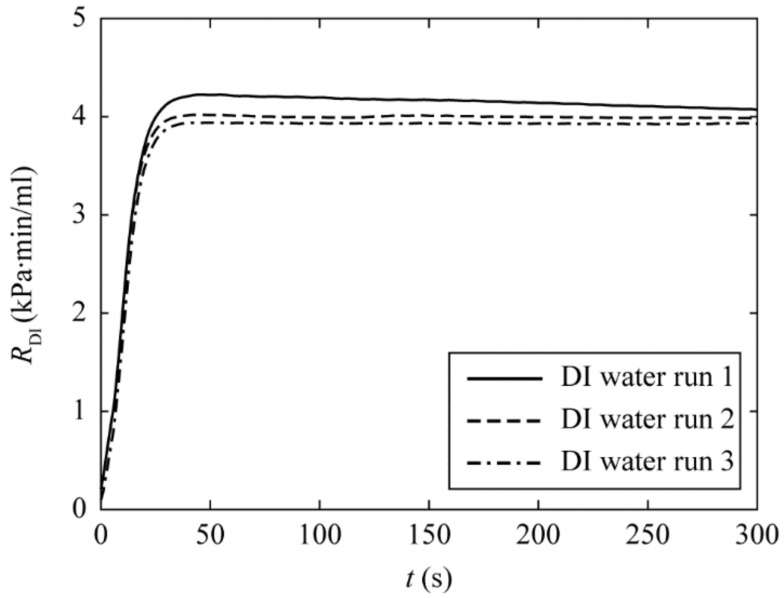
Plot of the hydraulic resistance for DI water runs, *R*_DI_, as a function of time. The data are sampled every second and plotted using thirteen points moving average. The solid, dashed and dash-dotted lines represent the hydraulic resistance of the filtration membrane in the 1st, 2nd and 3rd sequential runs, respectively. The difference in the steady-state *R*_DI_ for the last two runs is less than 2.0%. This implies that the filtration membrane is fully wet after the second DI water run, and can be used as the reference to be compared with in subsequent bacterial run.

Figure 2 shows the transient response of *R* for three sequential DI water runs. From these results, we make the following observations: first, the hydraulic resistance in the first run (solid line) overshoots to *R*_DI_ = 4.2 kPa⋅min/mL at the time *t* ≈ 50 s. During this time interval, we found that the color of the membrane filter turns from white to clear as it is gradually wetted by water. After that, *R*_DI_ gradually reduces to the steady-state value. This overshoot is not found in the subsequent two runs, and the pressure readings approach their steady-state values a lot faster. This could be due to the fact that the hydraulic property of the synthetic material composed of the filtration membrane is different before and after it is fully wetted by DI water. Second, the time required for *R*_DI_ to reach 95% steady-state value is about 12 s for the second and third DI water runs. It was also observed that the difference in the steady-state *R*_DI_ for the last two runs is less than 2.0% (compare the dashed and dash-dotted lines). This implies that after the filtration membrane is fully wet after second DI water runs, the steady-state pressure reading can be used to determine the hydraulic resistance of a filtration membrane. Therefore, the steady-state *R*_DI_ averaged from *t* = 150 s to 300 s in the second DI water is used to approximate the steady-state hydraulic resistance value, and serves as the reference to be compared with in subsequent bacterial run. As a side note, we quantified *R*_DI_ for the fourth and fifth DI water runs, and found that the steady-state readings show small fluctuations around the mean value (<2%, data not shown).

### 3.2. Calibration of the Differential Pressure and Hydraulic Resistance of Filtration Membrane due to Bacteria Trapping

After establishing the *R*_DI_ value of the syringe filter, bacteria are infused into the filter, and the transient response in the differential pressure is recorded by a manometer. Since the mean pore size of the membrane of 0.2 µm is much smaller than the main body size of *E. coli* (ellipsoidal shape of 1–2 µm in length and 0.5 µm in diameter based on our microscopic (Axio Observer.Z1, Carl Zeiss, Jena, Germany) observation), we expect that all infused bacteria are trapped. This is confirmed by sampling the filtered sample in the reservoir; no presence of bacteria is observed via direct microscopic observation. 

In order to compensate for the intrinsic differences in hydraulic resistance among the filtration membranes, we normalized time-dependent hydraulic resistance for bacterial run, *R*_Bact_, with the steady-state hydraulic resistance in the DI water run, *R*_DI,steady_, of that particular filter:

(2) $$\tilde{R}=\frac{R_{\mathrm{Bact}}}{R_{\mathrm{DI},\mathrm{steady}}}.$$

**Figure 3:**
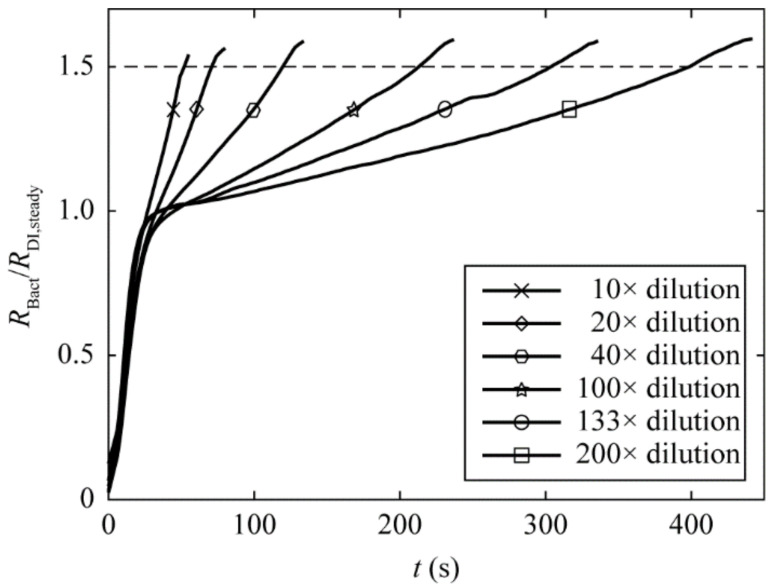
Plot of the hydraulic resistance due to bacteria trapping, normalized by the steady-state DI water hydraulic resistance, as a function of the measurement time, *t*, for six bacterial dilutions prepared at 10, 20, 40, 100, 133 and 200 times dilution factors from a same bacteria culture tube. As more and more micropores are blocked by trapped bacteria, the normalized hydraulic resistance is increased. We define the termination time, *t*_0_, as the infusion time required to reach normalized hydraulic resistance of 1.5 (as shown in the dashed line), and is used to reflect the number of bacteria trapped on the membrane, hence the bacterial density.

We then determined the change of the normalized hydraulic resistance as a function of time by infusing six bacterial solutions (Figure 3) diluted from a common stock culture with bacterial density of 2.12 × 10^9^ CFU/mL (determined from agar plate count). Figure S1 shows the plot of the hydraulic resistance due to bacteria trapping for nine additional independent sets of experiments. The densities of the bacterial samples typically range from 8.1 × 10^6^ CFU/mL to 2.2 × 10^8^ CFU/mL. However, due to the fact that bacterial stock solutions were cultured on different days, we expect that even at the same dilution factor, a large variation in the hydraulic resistance is observed among the 10 calibration sets (by comparing the plots among Figure 3 and Figure S1). 

The termination time, defined as the time required for $\tilde{R}$ to reach its threshold ${\tilde{R}}_{\mathrm{thres}}=1.5$ and denoted by *t*_0_, is used to determine the number of bacteria trapped on the membrane, and hence the bacterial density. The criterion ${\tilde{R}}_{\mathrm{thres}}=1.5$ is an optimized parameter that balances the sample-to-result time and the measurement error by allowing sufficient amount of bacteria to be trapped on the membrane. We did not test bacterial samples at higher density; we expect that the pressure difference curve raises extremely fast (less than 30 s) to pass the threshold value, and that we cannot distinguish whether the pressure increase is due to the transient response of the membrane filter (the fast-increase region of *R*_DI_ in Figure 2) or the bacterial disposition. At a bacterial density lower than 10^6^ CFU/mL, sample-to-result time was more than 1 h, which did not align with the rapid-detection aim. Within the working range of our device, we found that the hydraulic resistance in bacterial runs rises nonlinearly and becomes higher than that of the DI water run at *t* > 30 s. We postulate that when the individual bacterial cells are trapped at the membrane pores, the flow in the membrane filter has to be rearranged and that the bacterial deposition may cause a time lag in the increase in the pressure across the membrane filter. After that, the normalized hydraulic resistance further increases as more and more micropores are blocked by trapped bacteria.

### 3.3. Determination of the Bacterial Density Based on Termination Time

**Figure 4:**
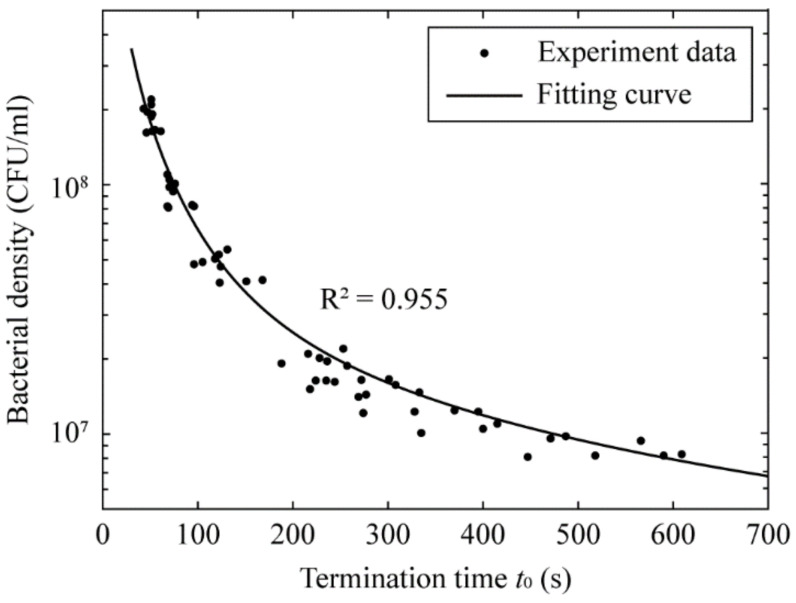
Semi-log plot of the bacterial density (based on agar plate counting) of 60 calibration samples prepared at various concentration ranging from 10^6^ to 10^8^ CFU/mL against their respective time taken to reach termination condition of ${\tilde{R}}_{\mathrm{thres}}=1.5$. It is evident that the bacterial density has an inverse relationship with the termination time. The 60 data points are fitted to Equation (7), resulting in the optimal theoretical curve with *R*^2^ = 0.955 (see the solid fitting curve).

Based on results from the parametric study with 60 calibration samples (ten separate sets of experiments, each with *E. coli* bacteria prepared at six different dilution factors), we set forth to determine the relationship between the bacterial density with termination time. To establish a fair analysis of the hydraulic resistances among the 10 calibration sets, in Figure 4 we plot the bacterial density of the inlet solution, determined by agar plate count, as a function of termination time *t*_0_. We found an inverse relationship between these two quantities: the higher the bacterial density in the infused sample, the lower the time required to reach the termination condition. When the bacterial density is in the order of 10^8^ CFU/mL (the most sensitive range of OD600 measurement, refer to fig. 4a of Ref. [27]), our device is capable of determining the bacterial density in less than 1 min after infusing the bacterial sample to the device. On the other hand, when the bacterial density is reduced to 10^6^ CFU/mL, our setup takes slightly longer to reach the termination condition (about 12 min). This demonstrates a large working range (about three orders of magnitude) and rapid-detection capability of our device. We note that our device should be able to detect the bacterial density at even lower orders of magnitude; however, the sample-to-result time becomes substantially longer (up to a few hours), which may limit its practical uses for rapid enumeration. 

### 3.4. Equivalent Electric-Circuit Model

An equivalent electric-circuit model was developed to formulate the general form of a calibration curve for the prediction of the bacterial density of a bacterial sample with sample termination time. Analogous to how an electric resistor regulates its voltage and current, the pores on the filtration membranes serves as hydraulic resistors, where the size and blockage condition of the filtration membrane determine the pressure difference across the two ends at a constant flow rate. The left panel in Figure 1b shows the equivalent electric circuit of our device. We first assume a total of *N* uniform-sized pores distributed over the surface of the membrane, and the resistance of each pore is denoted by *r*_DI_(*t*). We adopted a parallel connection of the resistances due to all pores, which gives the steady-state resistance of the membrane without bacterial deposition:

(3) $$R_{\mathrm{DI}}=\frac{r_{\mathrm{DI}}}{N}.$$

The infused bacteria are trapped on the membrane, blocking the pores and consequently increasing the resistance of these pores. We show the equivalent electric circuit due to trapped bacteria in the right panel of Figure 1b. As the size of bacteria is much larger than the pore size, we further assume that first, one bacterial cell on average blocks *m* pores, and the resistance of each blocked pore increases to *r*_Bact_. As a result, the overall resistance of the membrane, *R*_Bact_, can be related to the bacterial density (in the unit of CFU/mL), *c*, by using:

(4) $$\frac{1}{R_{\mathrm{Bact}}}=\frac{N-mcQt}{r_{\mathrm{DI}}}+\frac{mcQt}{r_{\mathrm{Bact}}}.$$

We further assume that the time-dependent responses of *r*_DI_ and *r*_Bact_ follow

(5) $$r_{\mathrm{DI}}=r_{0}\left[1-\exp\left(-t/\tau \right)\right]$$

$$r_{\mathrm{Bact}}=r_{b}\left[1-\exp\left(-t/\tau \right)\right]$$

respectively, with *r*_b_ > *r*_0_, where *r*_0_ (*r*_b_) is the averaged steady-state resistance of a pore without (with) bacterial deposition, *t* is the time in the unit of second, and the time constant 𝜏 takes into consideration the transient response of the pressure reading (as shown in Figure 2). We note that in our assumption, despite of the zero hydraulic resistance at *t* = 0, our model serves as a good approximation of the filter’s response in the time interval in which the increase in hydraulic resistance of the membrane filter is dominated by the bacterial deposition. The latter is the critical in determining the bacterial density.

Rearranging Equations (2)–(6) gives us the relationship between the bacterial density and time, expressed as

$$c=\frac{C_{0}\left[\left(\tilde{R}-1\right)+\exp\left(-t/\tau \right)\right]}{t}$$

where the constant $C_{0}=N/\left[mQ\tilde{R}\left(1-r_{0}/r_{b}\right)\right]$. At our prescribed threshold $\tilde{R}={\tilde{R}}_{\mathrm{thres}}=1.5$, we find that the coefficients *C*_0_ = 9.5 × 10^9^ CFU∙s/mL and *𝜏* = 61.5 s result in the optimal theoretical curve with *R*^2^ = 0.955 (see the solid line in Figure 4). We note that the relationship between the bacterial density and time in Equation (7) simplifies the calibration progress for the end user, in which the two constants, *C*_0_ and *𝜏*, can be obtained with a few rounds of agar plate count and termination-time determination of the device.

### 3.5. Blind Test and Proof of Concept for Bacteria Enumeration

Upon successful characterization of the relationship between the bacterial density with the termination time, we conducted two sets of experiments to validate the proposed empirical model and quantify the device accuracy. The bacterial density in the blind test sets typically ranges from 7.3 × 10^6^ CFU/mL to 1.9 × 10^8^ CFU/mL.

**Figure 5:**
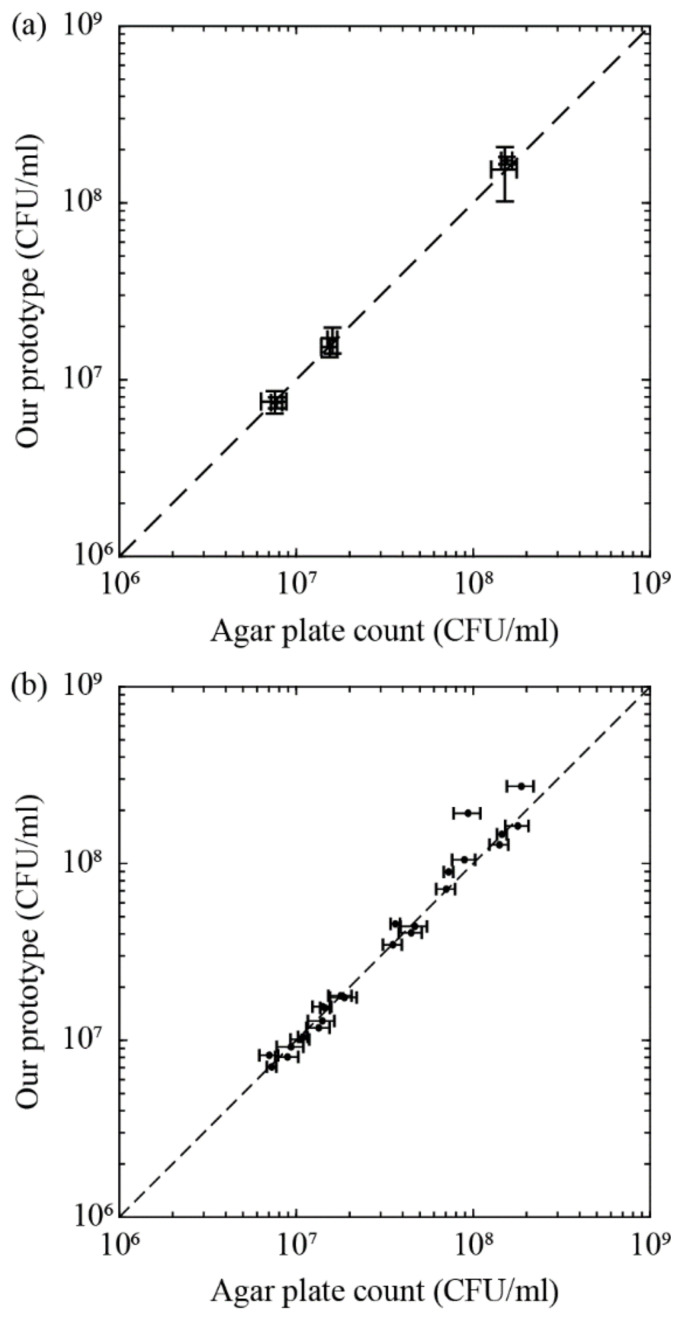
Proof of concept for bacteria enumeration. Log–log plots of the bacterial density determined from our prototype and agar plate count (**a**) for bacterial solutions of constant bacterial density to investigate the effects of intermembrane variations; and (**b**) to determine the averaged measurement accuracy of our device with 24 bacterial samples at various bacterial densities. The vertical and horizontal error bars in panel (**a**) represent the standard deviation for prototype and agar plate count, respectively. The error bars in panel (**b**) represent the standard deviation for agar plate count. The dashed lines in both panels are the reference lines indicating that the bacterial densities from both methods are identical.

In the first experiment set, we determined if the intermembrane variation plays a part on the bacterial density characterization by infusing samples of same bacterial density into a number of different filtration membranes. To achieve this, the stock *E. coli* solution was prepared using the same procedures as those in the calibration phase. Three serial dilutions were performed such that the bacterial densities of diluted solution covered the ‘lower’, ‘middle’ and ‘upper’ limits of the detectable range of our device. Each diluted solution was infused into the five different syringe filters, and the termination times determined by these experiments were substituted into Equation (7) to find the respective bacterial density. The experiments were performed twice. The conducted experiments are considered as blind tests, as the bacterial density was unknown on the day of the experiment and obtained through agar plate count the day after. Figure 5a plots the mean bacterial density and standard deviation for six bacterial solutions (two sets of experiments with ‘lower’, ‘middle’, and ‘upper’ limits), with each solution repeated with five separate syringe filters. We found that within the working range of our prototype, the mean bacterial densities quantified by our prototype agree well with those obtained from the agar plate count, and that the standard deviations from both methods are comparable. The raw data for the 30 runs are shown in Table S1 of the Supplementary Materials. The mean and median accuracies of bacterial densities obtained using these runs are 88.21% and 91.00%, respectively. Therefore, we conclude that the individual variation of the membranes has negligible contribution to the normalized hydraulic resistance and predicted bacterial density.

In the second experiment set, we determine the averaged measurement accuracy of our device with 24 *E. coli* bacterial samples of randomized densities, but all within the devices working range. Figure 5b shows the bacterial densities obtained by our prototype against the agar plate count for the 24 runs. The raw data for the 24 runs are shown in Table S2 of the Supplementary Materials. The results for most runs agree extremely well with those from the agar plate count, demonstrating that our device has the ability to predict the bacterial density in the order of 10^6^ to 10^8^ CFU/mL. The mean and median accuracies of bacterial density of the 24 runs are 85.95% and 91.50%, respectively.

### 3.6. Advantages and Improvements over Existing Methods

The two validation experiment sets elucidated the advantages of the proposed technique: automated, rapid and high accuracy. The end users only need to connect two syringes—one DI water and one bacterial sample—to the proposed setup. The device was then automated to fully wet the filtration membrane (which takes about 1 min), characterize the intrinsic hydraulic resistance of the wetted filtration membrane using DI water (about 5 min) and determine the bacterial density by substituting the termination time of the infused sample into the calibration curve. The total sample-to-result time is between 8 to 19 min, depending on the bacterial density. The overall mean and median accuracies of our device, derived from a total of 54 runs, are 87.21% and 91.33%, respectively.

In comparison to other low-cost bacterial enumeration techniques, particularly McFarland turbidity standards and spectrophotometer measurements, the consumables involved in the current setup are two syringes and a filtration membrane per run, which is estimated to cost USD 2 per measurement. The consumable cost is not much different from the other two techniques (about USD 0.2 per test). All techniques require users to independently calibrate microbes for use prior to determining microbial density, especially for microbes whose sizes are substantially different from the existing calibrations [26]. However, our proposed technique enables a total bacterial count of samples with bacterial densities of two orders of magnitude lower than the other two in reasonable sample-to-result time. In addition, our proposed technique mitigates subjectivity in interpreting the turbidity in conventional McFarland standards [49] and instrument and culture media dependencies in spectrophotometer measurements (refer to Refs. [26,50], Figure S2 and the “Effect of the solvent of bacterial solution on the OD600 reading” section in the Supplementary Materials); thus, offering a strong compelling alternative to the existing techniques in rapid determination of bacteria density using an inexpensive setup. The device can be further miniaturized and integrated with liquid metal or carbon microcoil for the pressure measurement [51,52].

Lastly, we point out that the viscosity of the solvent, which depends on solute concentrations and temperature, has minor effects on the differential pressure reading of our device. We demonstrate in the “Effect of solvent temperature and viscosity on differential pressure reading” section of the Supplementary Materials that when the solvent viscosity doubles, the differential pressure reading increases by at most 20% (Figure S3). Although nondimensionalizing the hydraulic resistance (Equation (2)) can partially compensate the viscosity effect, extra precaution should be made when using the one calibration curve to numerate the bacterial density for a sample with distinct viscosity.

## 4. Conclusions

In conclusion, we presented a rapid and low-cost bacterial-density enumeration technique through membrane filtration and differential pressure measurements. We determined the quantitative relative relationship between the bacterial density and time taken for the pressure difference across the membrane to reach the detection threshold. In addition, we demonstrated that the intermembrane variation has negligible contribution to the predicted bacterial density. The proposed technique is capable of determining bacterial density ranging from 7.3 × 10^6^ to 2.2 × 10^8^ CFU/mL with high accuracy.

## Data Availability

The data presented in this study are openly available online at doi:10.21979/N9/5EQO81.

## Supplementary Materials

The following supporting information can be downloaded at: https://www.mdpi.com/article/10.3390/mi13081198/s1, Figure S1: Plot of the hydraulic resistance due to bacteria trapping for 9 additional independent sets of experiments; Figure S2: Semi-log plot of OD600 reading as a function of *E. coli* bacterial density obtained from agar plate count in our experiment; Figure S3: Plot of the hydraulic resistance for DI water runs; Table S1: Six sets of raw data for Experiment 1; Table S2: Four sets of raw data for Experiment 2.
