# Supplementary material for: A Technique for Rapid Bacterial-Density Enumeration through Membrane Filtration and Differential Pressure Measurements
Source: Micromachines (Basel). 2022 Jul 28;13(8):1198. doi: 10.3390/mi13081198 (PMC9415702; doi:10.3390/mi13081198)
Supplement: Supplementary file 1 [file micromachines-13-01198-s001.zip › micromachines-1792942-supplementary.pdf]

micromachines

Supplementary Information

A Technique for Rapid Bacterial-Density Enumeration through Membrane filtration and Differential Pressure Measurements

Xinhui Shen †, Ting Wei Teo †, Tian Fook Kong and Marcos *

School of Mechanical and Aerospace Engineering, Nanyang Technological University, Singapore; shenxh@ntu.edu.sg (X.S.); teotw@ntu.edu.sg (T.W.T.); tianfook@ntu.edu.sg (T.F.K.)
† These authors contributed equally to this work.
* Correspondence: marcos@ntu.edu.sg

Hydraulic resistance in calibration runs

Figure S1 plots the hydraulic resistances of the membrane filter due to bacterial deposition (normalized by the steady-state hydraulic resistance in the DI water run), as a function time for 9 calibration experimental sets.

Derivation of the Bacterial Density Equation

Here we show the detail derivation of Equation 7 in the main text. First, we multiply both sides of Equation 4 by r_{Bact}. Rearrange the equation gives us

$$\frac{r_{\mathrm{DI}}}{R_{\mathrm{Bact}}} = N - \left(1 - \frac{r_{\mathrm{DI}}}{r_{\mathrm{Bact}}}\right) mcQt\,. \tag{S1}$$

We then substitute Equations 5 and 6 into Equation S1, and obtain

$$\frac{r_0\left[1-\exp\left(-t/\tau\right)\right]}{R_{\mathrm{Bact}}} = N - \left(1 - \frac{r_0}{r_b}\right) mcQt\,. \tag{S2}$$

Equations 3 and 5 implies that $R_{\mathrm{DI,steady}} = r_0/N$, which combining with Eq. 2 gives

$$\tilde{R} = \frac{NR_{\mathrm{Bact}}}{r_0}\,. \tag{S3}$$

We further substitute Equation S3 into Equation S2 and obtain

$$\frac{N\left[1-\exp\left(-t/\tau\right)\right]}{\tilde{R}} = N - \left(1 - \frac{r_0}{r_b}\right) mcQt\,. \tag{S4}$$

Lastly, rearranging Equation S4 gives us the bacterial density as a function of time

$$c = \frac{N}{\left(1-r_0/r_b\right) mQ\tilde{R}t}\left[\left(\tilde{R}-1\right)-\exp\left(-t/\tau\right)\right]. \tag{S5}$$

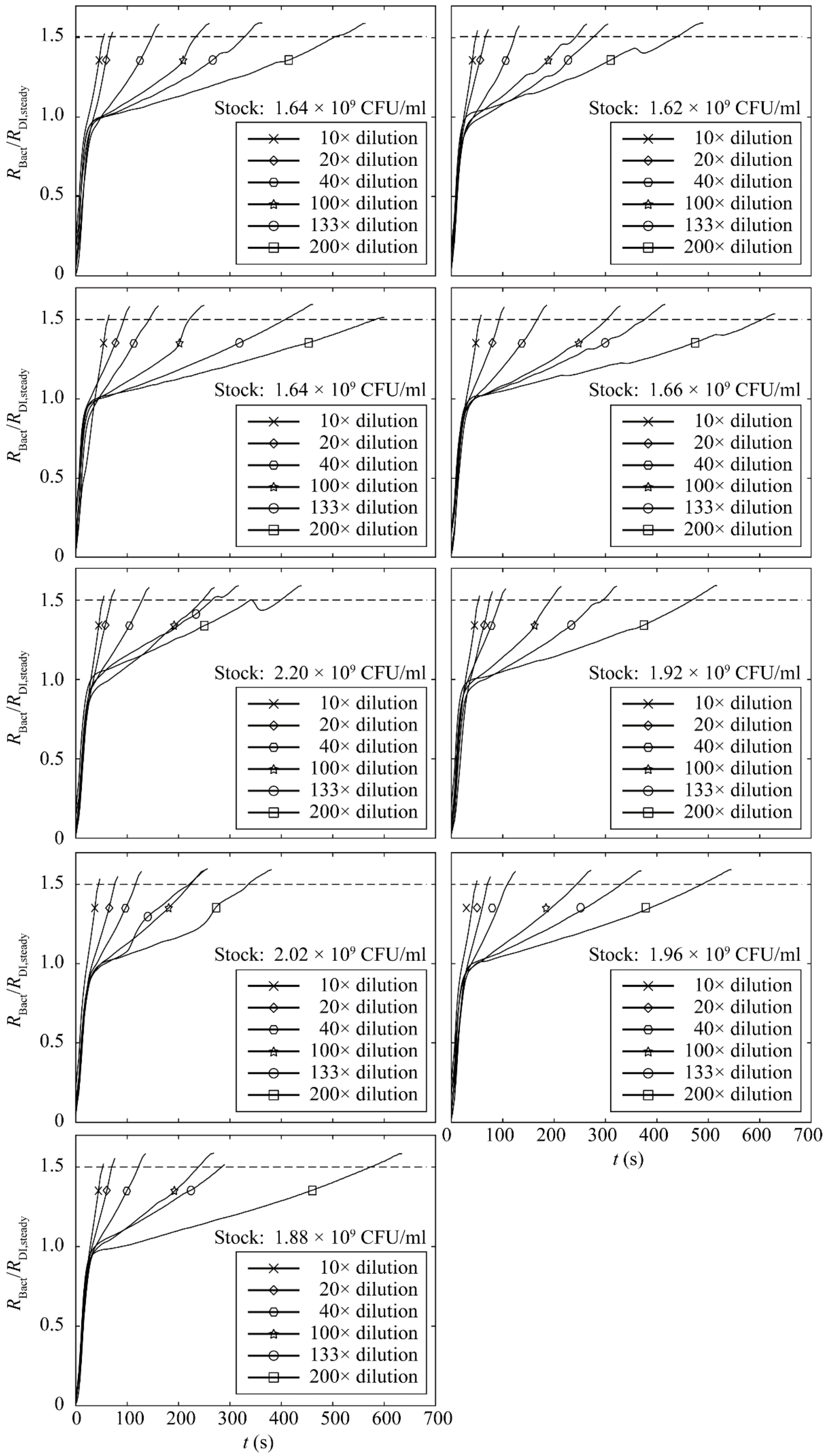

Figure S1. Plot of the hydraulic resistance due to bacteria trapping for 9 additional independent sets of experiments, normalized by the steady-state DI water hydraulic resistance, as a function of the

measurement time, t, for six bacterial dilutions prepared at 10, 20, 40, 100, 133, and 200× dilution factors from a same bacteria culture tube.

Raw Data for Blind Tests

Table S1. Six sets of raw data for Experiment 1. In each experiment set, samples of same bacterial density into a five filtration membranes.

Bacteria Density from Agar Plate Count (CFU/ml)	Termination Time (s)	Bacteria Density from the Prototype (CFU/ml)	Error
$(1.60 \pm 0.10) \times 10^7$	319.1	1.50×10^7	6.23
	312.6	1.54×10^7	4.19
	230.3	2.16×10^7	34.57
	278.2	1.74×10^7	8.68
	320.4	1.50×10^7	6.63
$(1.55 \pm 0.16) \times 10^7$	315.0	1.52×10^7	1.39
	307.8	1.56×10^7	1.06
	356.3	1.34×10^7	13.31
	278.2	1.74×10^7	12.74
$(1.51 \pm 0.25) \times 10^8$	283.6	1.71×10^7	10.42
	39.8	2.44×10^8	61.57
	60.2	1.38×10^8	8.57
	57.1	1.49×10^8	1.56
	62.0	1.33×10^8	12.33
	71.5	1.08×10^8	28.72
$(7.56 \pm 1.24) \times 10^6$	641.8	7.40×10^6	2.17
	620.7	7.65×10^6	1.15
	735.3	6.45×10^6	14.62
	694.3	6.84×10^6	9.58
	511.7	9.28×10^6	22.73
$(1.55 \pm 0.11) \times 10^8$	51.9	1.70×10^8	9.55
	53.3	1.64×10^8	5.50
	52.0	1.70×10^8	9.32
	49.1	1.83×10^8	18.19
	49.7	1.81×10^8	16.36
$(7.76 \pm 0.56) \times 10^6$	582.8	8.15×10^6	4.96
	641.9	7.39×10^6	4.72
	706.4	6.72×10^6	13.42
	624.1	7.60×10^6	2.00
	659.8	7.19×10^6	7.31

Table S2. Four sets of raw data for Experiment 2. In each experiment set, samples are diluted from a common stock culture and flow into a filtration membrane.

Bacteria Density from Agar Plate Count (CFU/ml)	Termination Time (s)	Bacteria Density from the Prototype (CFU/ml)	Error
$(1.79 \pm 0.27) \times 10^8$	53.5	1.63 × 108	8.67
$(8.93 \pm 1.33) \times 10^7$	72.8	1.05 × 108	17.67
$(4.47 \pm 0.67) \times 10^7$	140.7	4.06 × 107	9.10
$(1.79 \pm 0.27) \times 10^7$	272.2	1.79 × 107	0.04
$(1.34 \pm 0.20) \times 10^6$	404.9	1.18 × 107	12.25
$(8.93 \pm 1.33) \times 10^6$	590.1	8.04 × 106	9.93
$(1.87 \pm 0.32) \times 10^8$	36.5	2.73 × 108	46.02
$(9.36 \pm 1.60) \times 10^7$	47.4	1.93 × 108	105.81

$(4.68 \pm 0.80) \times 10^7$	132.5	4.41 × 107	5.67
$(1.87 \pm 0.32) \times 10^7$	277.7	1.75 × 107	6.71
$(1.40 \pm 0.24) \times 10^7$	370.5	1.29 × 107	8.33
$(9.36 \pm 1.60) \times 10^6$	517.1	9.18 × 106	1.90
$(1.41 \pm 0.17) \times 10^8$	63.6	1.28 × 108	9.35
$(7.04 \pm 0.86) \times 10^7$	94.7	7.16 × 107	1.74
$(3.52 \pm 0.43) \times 10^7$	157.7	3.47 × 107	1.35
$(1.41 \pm 0.72) \times 10^7$	310	1.55 × 107	10.12
$(1.06 \pm 0.13) \times 10^7$	469.7	1.01 × 107	4.21
$(7.04 \pm 0.86) \times 10^6$	576.7	8.23 × 106	16.92
$(1.45 \pm 0.09) \times 10^8$	57.7	1.47 × 108	0.78
$(7.27 \pm 0.45) \times 10^7$	81.2	8.97 × 107	23.42
$(3.64 \pm 0.22) \times 10^7$	129.5	4.56 × 107	25.41
$(1.45 \pm 0.09) \times 10^7$	315	1.52 × 107	4.87
$(1.09 \pm 0.07) \times 10^7$	454.8	1.04 × 107	4.18
$(7.27 \pm 0.45) \times 10^6$	671.2	7.07 × 106	2.74

Effect of the Solvent of Bacterial Solution on the OD600 Reading

Here we demonstrate the effect of liquid media on the OD600 reading. We conduct an additional experiment which first cultures an *E. coli* stock solution using the culturing technique as stated in the manuscript. We then divide the solution into two samples of equal volume, one diluted with clear culture media and the other with DI water. The samples at various dilution factors are measured using a spectrophotometer (Unico Spectrophotometer S-1000E). The exact *E. coli* density is determined based on the agar plate count as stated in the manuscript. Figure S2 shows the OD600 reading as a function of bacterial density obtained from the agar plate count on the next day of our experiment. We find that the OD600 measurements are insensitive to the bacterial density at 10^7 CFU/ml, and that the choice of solvents significantly affects the OD600 results.

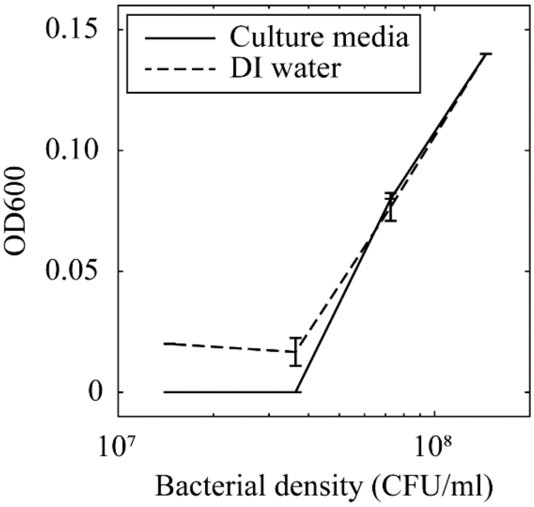

Figure S2. Semilog plot of OD600 reading as a function of *E. coli* bacterial density obtained from agar plate count in our experiment. The solid and dotted lines represent the OD600 reading when the dilution media is culture media and DI water, respectively. The error bar presents the standard deviation of three measurements.

Effect of Solvent Temperature and Viscosity on Differential Pressure Reading

We note that the experiment conducted in the room temperature (25 °C), in which the viscosity of DI water is 8.9×10^{-4} Pa·s, and that the water viscosity at 0 °C increases to 1.8×10^{-3} Pa·s. Therefore, one may concern that the differential pressure reading across

one membrane filter may be significantly affected by the solvent temperature and viscosity.

Here we quantify the differential pressure reading when DI water at 0 °C and 25 °C. We first fully wet a membrane filter using the DI water at room temperature. After that, we infuse the room temperature DI water in again at the flow rate of 3 ml/min and obtain a steady-state pressure difference reading across the membrane filter, ΔP_{DI}. Lastly, we infuse DI water drawn from the ice-water mixture (0 °C) into the same membrane filter for three times, and measure ΔP_{DI} again in the consecutive runs.

Figure S3 shows the pressure differences as a function of time for the DI water runs at two different temperatures. We find that the hydraulic resistance at 0 °C is at most 20% higher than that at 25 °C, even though the dynamic viscosity of the former is doubles the latter (compare the solid grey and black lines in Figure S3). In comparison, the standard deviation of the hydraulic resistance of filter individuals is 14% (3.6 ± 0.5 kPa·min/ml, mean ± sd). The hydraulic resistances of the second and third 0 °C DI water runs decrease due to the fact that the water temperature slowly raises when we conduct the experiment in about 20 mins. Therefore, the media viscosity has only minor effect on the pressure reading, especially for the case that the sample temperature and viscosity do not vary much from those during the calibration runs.

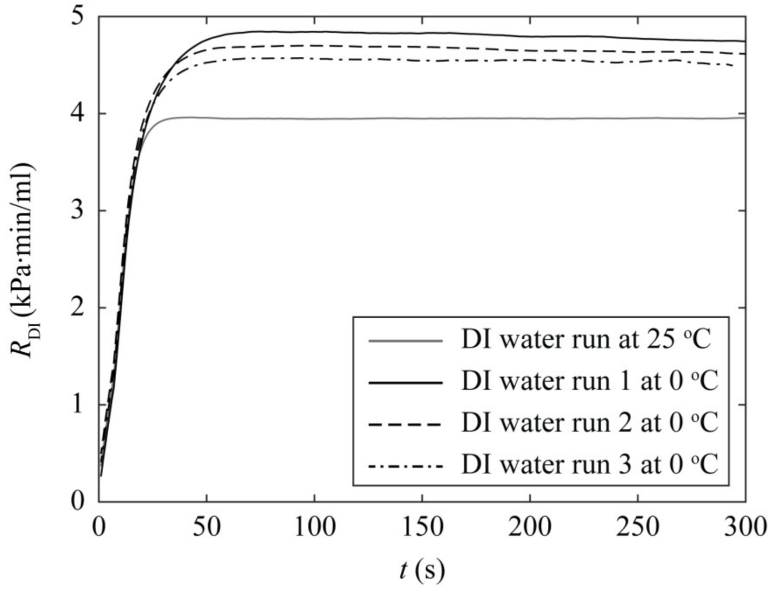

Figure S3. Plot of the hydraulic resistance for DI water runs, R_{DI}, at two different temperatures (0 and 25 °C) as a function of time. The data is sampled every second and plotted using thirteen points moving average.
